# Navigating environmental constraints to injection preparation: the use of saliva and other alternatives to sterile water among unstably housed PWID in London

**DOI:** 10.1186/s12954-020-00369-0

**Published:** 2020-04-10

**Authors:** Magdalena Harris, Jenny Scott, Vivian Hope, Talen Wright, Catherine McGowan, Daniel Ciccarone

**Affiliations:** 1 Department of Public Health, Environments and Society, London School of Hygiene & Tropical Medicine, 15-17 Tavistock Place, London, WC1H 9SH UK; 2 Department of Pharmacy & Pharmacology, University of Bath, Claverton Down, Bath, BA2 7AY UK; 3 Public Health Institute, Liverpool John Moores University, Tithebarn Street, Liverpool, L2 2QP UK; 4 School of Medicine, University of California, San Francisco, 513 Parnassus Ave, San Francisco, CA 94143-0410 USA

**Keywords:** Water for injection, Injection preparation, People who inject drugs, Homelessness, Skin and soft tissue infections, Crack cocaine, heroin

## Abstract

**Background:**

The United Kingdom is experiencing an increase in drug-related deaths and serious bacterial infections among its most vulnerable citizens. Cuts to essential services, coupled with a growing homeless population, create a challenging environment to tackle this public health crisis. In this paper, we highlight an underexplored environmental constraint faced by people living and injecting drugs on the streets. Access to water for injection is restricted in the UK, due to legislative and financial barriers. Austerity measures, such as public toilet closures, further restrict the ability of people made homeless to access clean water and protect themselves from health harms.

**Methods:**

We generated questionnaire (*n* = 455) and in-depth qualitative interview (*n* = 32) data with people who inject drugs in London for the Care and Prevent study. Participants provided detail on their life history; drug use, injecting and living environments; health conditions and care seeking practices.

**Findings:**

A high proportion of the survey sample reported lifetime history of street homelessness (78%), bacterial infections (65%) and related hospitalisation (30%). Qualitative accounts highlight unsafe, potentially dangerous, injection practices in semi-public spaces. Multiple constraints to sourcing sterile water for injection preparation were reported. Alternatives to sterile water included puddle water, toilet cistern water, whisky, cola soda and saliva. Participants who injected heroin and crack cocaine together unanimously reported adding water at two stages during injection preparation: first, adding water as a vehicle for heroin (which was then heated); second, adding cold water to the heroin mixture prior to adding the crack cocaine. This new finding of a stage addition of solvent may represent an additional risk of infection.

**Conclusion:**

Currently, harm reduction equipment and resources for safe injecting are not meeting the needs of people who inject drugs who are street homeless or unstably housed. Preparation of injections with non-sterile water sources could precipitate bacterial and fungal infections, particularly when used without the application of heat. It is crucial that water for injection, also skin cleaning, is made available for the unstably housed and that harm reduction messaging is tailored to speak to the everyday realities of people who prepare and inject drugs in public spaces.

## Background

Health harms are increasing among the estimated 200,000 people who inject drugs (PWID) in the United Kingdom (UK). Since 2012, there has been a steady increase in hospitalisations for serious bacterial infections [1], a doubling of opioid-related deaths [2], and outbreaks of invasive and infectious diseases among PWID, particularly among those who are homeless [1–3]. The recent outbreak of coronavirus (COVID-19) is of particular concern, given vulnerability to respiratory problems among PWID in the UK, many of whom also smoke (crack cocaine, heroin, tobacco) and have a much higher incidence of chronic obstructive pulmonary disease (COPD) than the general population [4]. Drug-related deaths in the UK are higher than any European Union (EU) country; in the year before Britians exit they accounted for one third of all reported in the EU country and, in the year prior to Britain’s exit, accounted for a third of all reported in the EU [5]. In an austerity context, where health harms are exacerbated by cuts to social and health services [6]—particularly among a growing homeless population [7]—services must innovate to reach those most at risk. To do so effectively, it is essential that interventions are informed by an in-depth understanding of current constraints to safe injecting practices and how environments can be modified to facilitate or protect against health harms.

Homelessness and public injecting are interrelated and constitute part of the structural risk environment fostering health harms, such as bacterial and viral infections, among PWID [8]. Skin and soft tissue infections (SSTI), such as abscesses and cellulitis, are a growing cause of hospitalisation among PWID [9], with homeless PWID particularly susceptible to SSTI and related complications, such as septicaemia and endocarditis [10]. This increased risk among the unstably housed may be a consequence of inability to maintain safe and hygienic injection practices when injecting in public places, where injections may be hurried, with a lack of clean surfaces, poor lighting and difficulties in accessing clean water to use as a drug solvent [11–15]. The use of water from toilets, cisterns, ditches and puddles has been noted [11, 15–23], and these sources are likely to be contaminated with particulates and microorganisms. Although few studies have specifically explored the role of water used to prepare injections in relation to SSTI among PWID, there are indications that the use of puddle and toilet water is a risk [20, 21]. Indications of difficulty accessing water among the most marginalised are of crucial concern also, given the potentially protective benefit hand washing can have against viral infections such as influenza and COVID-19.

Water is required to prepare street drugs such as heroin and crack cocaine into a solution for injection. As a component of injection preparation, water is a concern due to its ability to carry micro-organisms, and as it might be shared when groups of people inject drugs together. In the UK, “water for injection” (WFI) is a sterile pharmaceutical product used to reconstitute medicinal injections. It has to meet a range of quality standards including limits on particulate contamination and bacterial endotoxins [24]. Prior to 2003, supply of WFI to PWID was prohibited in the UK under the 1971 Misuse of Drugs Act. In response to the increased evidence of hepatitis C (HCV) risk from sharing injecting paraphernalia (i.e. injecting equipment other than needles and syringes) [25, 26], section 9A of the Act was amended in 2003 to decriminalise the supply of WFI and other paraphernalia by practitioners, pharmacists and people engaged in the “lawful provision of drug treatment services” [27]. Under the Medicines Act 1968, however, all medicines for parenteral administration, including WFI, are classified as a prescription only medicine [28]. This prevented needle and syringe programmes (NSP) effectively supplying WFI, despite the 2003 law change, unless it was issued under prescription. Contraventions to the law during this time included the supply, by a social enterprise, of plastic 1.4 ml sterile water ampoules. Available from September 2003, supply was halted in June 2004 by the Medicines and Healthcare Regulatory Authority (MHRA), as the ampoules were deemed by the MHRA “to have the appearance of a medicine”, and thus illegal to distribute without a medicines license [29]. A campaign ensued to change the status of WFI from prescription only medicine to enable its supply though NSP. In June 2005, this was realised; with the regulations amended so that drug treatment services (including NSP) could supply WFI to PWID [30]. The status of WFI as a prescription only medicine remains however, with a prescription required for all other uses other than supply to PWID.

A maximum 2 ml volume restriction was initially placed on ampoules of WFI supplied to PWID, in order to reduce potential for water sharing [28]. This concern was largely related to HCV risk, given evidence that HCV can survive for up to 3 weeks in bottled water and on water containers after use [25]. There was a further amendment of the Medicines Act in 2012 to allow the supply of 5 ml water ampoules by drug workers to PWID, but with no public announcement to this effect, awareness of the change among injecting equipment manufacturers and providers was minimal [31]. The only 2 ml licensed and marketed WFI available in the UK is in a glass ampoule. A “safe snap” ampoule snapper was made available in 2006 [23, 29] to reduce the risk of glass cuts and associated HCV transmission potential. Some NSP remained reluctant to provide glass ampoules due to concerns about ampoule cost or the percieved potential for glass cuts if opened without an ampoule snapper [23]. Distribution of 5 ml ampoules of water is limited again due to cost, and also ignorance of the change in the law, drug litter potential, and fears of HCV infection through shared ampoules. At present, in the UK, provision of WFI through NSP is limited, fragmented and inconsistent. The majority of injecting supplies are now accessed through community pharmacies, which usually dispense standard issue injecting packs, and these rarely contain WFI.

There is a need to interrogate current harm reduction messaging and injecting paraphernalia provision to ascertain whether it meets the needs of PWID who are rough sleeping or unstably housed. In the UK, this is a growing population, increasingly vulnerable to health harms. Relatively little research has focused on the use of water for illicit drug preparations, apart from assessing viral transmission risk. Current harm reduction advice advocates the use of WFI, with the “next best” alternative boiled and cooled potable (drinkable cold tap) water, and after this, cold tap water. Our research illustrates that while these hierarchies of best practice may be actionable by people living and injecting in homes, they are not necessarily feasible for people living or injecting on the streets. We present mixed-method data generated with PWID in London to highlight the environmental constraints faced by people living and injecting drugs on the streets, with a focus here on reports of liquids used for drug preparation purposes. In doing so, we aim to inform pragmatic interventions to reduce drug, and other related, health harms among the most marginalised PWID in the UK.

## Methods

This paper reports on data from the London-based, National Institute for Health Research funded, Care and Prevent Study. This mixed method study generated questionnaire with urinalysis (*n* = 455) and qualitative interview (*n* = 32) data with PWID in London, UK, between October 2017 and March 2019. The aim of Care & Prevent is twofold: (1) to explore associations between SSTI and the renal disease, AA amyloidosis, including though risk screening and specialist referral and (2) to explore the risk factors and contexts related to SSTI among PWID. This paper will present findings pertaining to the latter aim, primarily drawing on qualitative accounts of injection preparation practices.

Study methods have been published in detail [32]. Participants were recruited through word of mouth and recruitment flyers at drug treatment services, homeless hostels and day centres across London. Eligibility criteria comprised the following: ability to provide informed consent, aged 18 years or over and having a history of psychoactive injecting drug use. Surveys, urinalysis and qualitative interviews primarily took place at private rooms in the recruiting services, with some qualitative interviews conducted in a café, on the street or in participants’ homes. All were reimbursed a £10 voucher for the survey and urinalysis and £20 for an interview. The questionnaire was entered into Open Data Kit (ODK) software and researcher administered using the ODK Collect application on Android tablets [33]. Participants provided information on their socio-demographics, drug use history, injection preparation and administration practices, equipment use and reuse, health care practices, lifetime experience of SSTI and other health conditions. Qualitative interviews sought detail on areas outlined above, were of 60–120 min duration, audio-recorded with consent and professionally transcribed verbatim.

Using Stata version 15.1, the characteristics of study population were described using numbers and percentages for categorical variables, means (standard deviations) for normally distributed continuous variables and median (95% CI) for non-normally distributed continuous variables. No multivariate analyses were undertaken for this paper as the risk variable, water, was not measured. Qualitative analysis was informed by constructivist grounded theory methods [34] with data analysed as generated in order to inform the direction of subsequent interviews, coding, case selection, memo and theory generation. A selection of interviews was open coded using process or gerund codes [34]. Consolidation of these codes formed the basis of a coding frame, against which all transcripts were coded (first stage). Second stage coding comprised inductive open coding of data in each category to inform theme development and interpretation for publication.

Ethical approval was granted by the London School of Hygiene and Tropical Medicine Observational Research Ethics Committee [12021], the London Bridge Research Ethics Committee and Health Research Authority [17/LO/0872]. All participants provided written consent after receiving study information and assurance of confidentiality.

## Results

### Survey

Three quarters of those participating the survey (75%, 341/455) identified as male and (74%, *n* = 336) as white or white British. Participants’ mean age was 46 years (range 21 to 68). Most (79%, *n* = 360) were currently receiving opioid agonist therapy, with two thirds (62%, *n* = 284) reporting drug injecting in the past 12 months. Use of crack and heroin in combination, aka “snowball”, was favoured, with 49% (*n* = 255) reporting this as their primary drug injected, risng to 58% among those who had injected in the past year (*n* = 164/284). Of note, given the vulnerability to COVID-19 among those with respiratory problems, 61% (*n* = 278) of the sample reported current crack smoking, 47% (*n* = 214) current heroin smoking, and 91% (*n* = 414) current tobacco smoking, the latter for a median duration of 29 years. Sixty-four (14%) participants had received a diagnosis of COPD, with a number likely undiagnosed. Most participants (78%, *n* = 355) reported a lifetime history of street homelessness with a mean duration of four years (range < 1 to 30 years). Just over half (57%, *n* = 259) were unstably housed for all or most of the past 12 months, with 33% (*n* = 152) in hostels, 14% (*n* = 61) street homeless, 7% (*n* = 31) staying with friends or family and 3% (*n* = 15) in prison. Participants were asked about lifetime experience of abscesses, cellulitis, venous ulcers and venous disease. The majority (65%, *n* = 296) had experienced at least one of these conditions, of whom 46% (*n* = 137) reported consequential hospitalisation. Notably, of the 291 participants (64%) reporting a bacterial infection (abscess or cellulitis), 27% (*n* = 80) reported a diagnosis of septicaemia and 7% (*n* = 21) endocarditis.

### Qualitative findings

Qualitative interview participants (*n* = 32) had similar characteristics to the survey sample, with 69% (*n* = 22) male; 81% (*n* = 26) white or white British, average age of 43 years old and 94% (*n* = 30) had experienced homelessness. Qualitative accounts indicate how injecting preparation practices, and the environments in which they are situated, can facilitate risk for SSTI and serious sequelae. Below, we present data pertaining to the injection preparation process with a focus on water source as an environmental constraint to safe practice and the strategies employed by participants to navigate this constraint.

#### “Snowball” injection preparation requires “cooler”

Participants were asked to describe in detail their process for preparing a “typical injection”. All participants followed a similar procedure for preparing heroin, with variation in relation to amount of acidifier used and the order in which acidifier, heroin and a solvent were combined in the spoon (for more information on this process, see [35]). This mixture was then heated, prior to drawing up through a filter ready for injection. Those who injected heroin and crack cocaine in combination all referred to the addition of cold water (or other cold solvent) prior to adding the crack. The proportion of cold water or “cooler” added to the heroin mix was detailed precisely and appeared remarkably uniform across accounts. Troy and Tim’s accounts are indicative:

The citric goes into the spoon first, I always inject probably 100mls^1^ of water, or like suck in 100mls water … I just put the brown in and cook up the brown, put 80mils of water in, then 20mils of cooler and then the crack, squash it up and use a filter.

So, you don’t heat the crack do you?

No, I heat the brown and then I put a cooler in. Yeah, like 20mils of water, yeah. (Troy)

Yeah, put the gear in, burn it up, cook it up … put water in and then put citric in … and cook, burn the brown up, then put about 20mil cooler and then put the white in, and just crush the white up, and draw it up and bang it. (Tim)

While Ross and Lee do not specify the amount of cooler used, or refer to it in those terms, their method follows a similar process:

I’d put a little bit of citric in the spoon, open the stuff [heroin], put it in, add the water, cook it, put it down, add the rest of the water, put the white [crack] in, crush it up properly, put the filter in, pull it up. (Lee)

I would put in two bags of heroin, so maybe like .4 [unit of street drug deal] and I’d then put in .2 of crack. No, first I would put in .4 of heroin, maybe 1 citric [sachet] for £20 worth of heroin, I would cook that up, I’d then add a little bit of cold water and put £10 worth of crack in there, make sure I can’t see it anymore, although it’s hard because it’s quite shit and then I’d suck it up, usually using the 2 ml [syringe]. (Ross)

Ross’ reference to “quite shit” is echoed by others interviewed, with a general perspective that the quality of the crack available through London street markets at the time of the study was poor. When the end-point of preparation was judged by clarity of the injection solution, this could result in the addition of excessive acidifier to break down cutting agents and manufacturing by-products in the crack [35]. What these accounts also highlight, however, is that a proportion of the drug injection solution has not been subjected to heat, with implications for health harms depending on the contamination present in the “water” sourced.

#### Desperate constraints: sourcing water alternatives

The environments in which participants prepared injections posed multiple constraints to enacting “best practice” harm reduction advice. As noted above, nearly 80% of the survey sample reported a lifetime history of rough sleeping, rising to 94% of those providing qualitative accounts. Many interview participants spoke of their “typical injection” preparation processes occurring in public or semi-public settings such as public toilets, bin sheds, stairwells, car parks, parked cars and even moving buses. As detailed in other qualitative studies [11, 13–15], semi-public injecting is characterised by opportunity, urgency and constraint. Ben refers to the need for haste when injecting in semi-public settings “because you’re scared the police are going to come, or someone’s going to come”. When asked how this can impact on the injection preparation practice he replies “people might forget to put citric in or use puddle water”. While he is fearful to do this himself, he has seen others use puddle water “a lot”.

When asking about injection preparation practices, the first author took care to elicit specific detail about type and amount of acidifier used, but did not think to lend the same scrutiny to water source and type. Unelicited accounts of solvents used in the preparation process unsettled this lack of attention. Jeff, for example, spoke at length about the embodied effects of using cola as a water alternative, of which this quote is an extract:

I wouldn’t care where I cooked up, I’d cook up on rain water off the cars, car bonnets. I’ve even cooked up on Coca-Cola. Coca-Cola, yeah, and when you inject it you can taste it more, you can feel the coke going down the back of your throat, you feel like you’ve had a sip of coke. (Jeff)

Dean spoke of injections that “sting like fuck” when using “neat lemon juice” in place of water, acknowledging that he was “destroying my veins” by doing so. The use of alcohol in place of water was also frequently mentioned, and given that many rough sleeping participants were also heavy drinkers, this was likely to be a readily accessible liquid for injection preparation on the street:

I’ve used whisky, Tennent’s Super Lager, all sorts to do my hit … vodka, whisky, you name it, I’ve used alcohol to do my cooking, to do my [hit] (Neil)

These practices were not spoken of lightly. The need to source an alternative to water was a desperate constraint in the context of injection preparation. As Alex indicates: “I’ve used alcohol or fucking, yeah, man, you do things when you’re desperate, you know.”

The expression of desperation was most acute when participants spoke of using saliva as an alternative to water. For those who did so, this was framed as a last resort:

I was that desperate one time I was even trying to cook up with my own spit, that’s how desperate I was. [Did it work?] No, it was fucked, it wouldn’t cook, just frazzled … I was going to try it but when I seen it’s frazzled I said fuck it and I flung it away (Jeff)

Jeff was lucky. Although he lost the hit he had been hoping for, he did not experience the health harms recounted by Dev in conversation with the first author:

**You said you’ve got a wicked scar on your groin, right?**

*It’s infection, it got infected. What it was, there was no water actually and I had to use a bit of saliva. I had to use a bit of saliva, there was more gear, yeah.*

**There was no water, so you used saliva?**

*Yeah, thinking that could work and it didn’t work, man. It worked, I still got my hit, but I also got the worst infection of my life, I nearly died … Yeah, I was in hospital for nearly 3 months. Septicaemia.*

**Where were you cooking up the hit?**

*In the flats, bottom of the flats.***[In a stairwell?]***Yeah, I was homeless at this time, innit.*

**Can you tell me about it in detail, like walk me through what **happened?

*What I remember, I must have gone out, graft, scored, I didn’t have enough fucking [water] … I didn’t have no trouble getting my groin, I must have got it and then it, I had some sort of fever like fucking hell, fever come across me and it was bad so I had to go to hospital … after that I was knocked out then woke up in the hospital bed, I went for fucking surgery. And then a second surgery after that because they didn’t get all the infection out.*

**What did they say it was? Like, how did it get that bad that quickly?**

*It was an infection, an infection was found, a bug was found in the mouth … Yeah, in any mouth, in people’s mouths. Just that bug and it’s a nasty bug, a right nasty bug. And that’s why I remember when I tried to put a bit of saliva in, know what I mean, so, it makes sense.*

**So, was there was nowhere else you could get water from, or …?**

*No, no.*

#### Navigating constraint: trying to stay safe

The context of constraint for many was one where public access to water, for drinking, washing or other purposes was not guaranteed. This has implications not only for injecting related practices but also for the ability to stay hydrated, as Jeff mentions in passing below:

*We were on the streets, I can’t remember where I was, but there was no water about anywhere and it was a red-hot day as well, there was no water anywhere. And I was dehydrating, dehydrating, plus there’s no toilet, no taps anywhere around, no rain, no nothing. I was proper sick for a hit, in the end because I couldn’t find no water and my spit wouldn’t work and then I got another bag and I had to do it with a puddle of water and then like filter the water through the sterits [alcohol swab], filter it out that way.*

**Through the alcohol swabs?**

*Yeah, just to get the dirt of the water.*

Of note in this excerpt are the lengths that Jeff went to, not only to have a hit, but to navigate the constraints of his environment. Filtering puddle water through an alcohol swab (provided by NSP for wiping injecting sites) is a poignant example of self-care, also illustrating the short-sighted limitations of service provision where swabs are deemed essential but water ampoules are not. As Sara says “you don’t get water with the [injecting] kit.”

For Sara, and some other unstably housed participants, the answer is to carry bottled water. As Troy says: “I usually have a bottle with me, a bottle of water, yeah” and Jade: “then I would just draw up some water, because I have a bottle of water or something”. Ben details his rationale for using bottled water and his strategy for obtaining it:

*A lot of the time people would use puddle water. That’s a thing, yeah. But I was always scared of that, so I always got scared in case there was like an animal and then you’d suck the animal in and then you’d put the animal inside yourself … I used to always get people to buy me bottles of Volvic water and then I’d pull up from the bottle. People would come and buy me drinks and food all the time. They always asked me, what do you want from the shop?*

**Okay, so like people walking by on the street?**

*Yeah.*

**Yeah. So you’d specifically ask for bottled water so you could use it for having a hit?**

*Yeah. Yeah-yeah-yeah. (Ben)*

Participants also spoke of carrying baby wipes and swabs with them, particularly useful to wipe hands and injecting sites in the absence of water to wash with. Neil, who had prepared injections with a variety of alcoholic beverages, spoke with pride of keeping his injecting sites clean on the streets, showing the first author his stash of alcohol swabs, always replenished in his coat pocket: “I always have alcohol swabs and wipes and all that, just wee things like that, I always keep one … I’m just a survivor, love”. Lee would also carry baby wipes and bottled water with him when possible. He prioritised finding toilets to inject in that were “clean” and where he could wash his hands; however, bottled water (or an empty bottle) was often necessary, given the difficulty of obtaining water with a syringe from a public access sink when preparing and administering an injection in a closed toilet cubicle.

## Discussion

A well-established body of literature evidences health harms and health system burden associated with injecting drug use [36, 37]. UK surveillance data illustrate an increase in mortality and morbidity among PWID over the past decade [5, 38] particularly in areas of socio-economic deprivation [38] and those hit hardest by local authority funding cuts [39]. In the decade since the implementation of UK government fiscal austerity policies (2010–2020), it is estimated that local authorities will have faced a core funding reduction of £16 billion compared to the preceding decade [40]. These cuts have impacted not only on housing and welfare provision but access to clean water on the city streets. As the homeless population has increased in the UK, most notably in London,^2^ access to public amenities have decreased, with 673 public toilets closed by major councils since 2010 [42]. At the same time, drug treatment services, facing sustained budgets cuts of at least 18% [39], have reduced costs where possible, impacting on the availability of water provision in NSP equipment packs. The impact of these structural changes on injecting environments and practice is reflected in our qualitative accounts, with participants describing injection preparation with solvents ranging from puddle water to whisky to saliva when injecting in public and semi-public spaces.

The risks or health harms associated with use of alternative solvents for injection has not, to date, been elucidated in the literature, apart from reference to bacterial infections arising from saliva contaminated injections. While some harms are proven, many are theoretical and not easily measured. This is particularly the case in relation to use of alternatives to water. Alcohol for example, although an antibacterial and effective solvent for base drugs such as heroin, as an injection solvent may pose an overdose risk particularly in combination with heroin, methadone or other sedatives. Although the amount of alcohol injected is small, when used intravenously, it does not go through the liver before the brain, as it would with oral (drinking) administration; therefore, it reaches the brain faster and in a more concentrated form. Puddle water may contain a range of organisms found in the environment, including the heat resistant spores of anaerobic bacteria including *Clostridium* species that cause tetanus and botulism [43] and *Pseudomonas* species, associated with multidrug resistance and sepsis [21], as well as particulate matter and environmental chemicals. Saliva is a polymicrobial fluid. While heating drug solutions containing saliva may inhibit bacterial growth, not all organisms are likely to die at the same temperature, the same solution acidity or in such a short duration of heating time. Other components of saliva, such as proteins, may prompt an immune response if injected. The potential for infection from saliva injections is therefore highlighted as a concern.

Our study is the first to report, to our knowledge, purposeful use of saliva as an alternative to water in injection preparation. Bacterial infections attributed to saliva-contaminated injections have been documented in the literature, however, only in relation to the licking of needles and/or injection sites. Binswanger et al. [44] found that of 169 community-recruited PWID in San Francisco, 28% (*n* = 46) reported licking their needles prior to injection. On examination, 32% (*n* = 54) presented with abscesses and/or cellulitis. After controlling for skin-popping and years of injection drug use, participants who licked their needles were more likely to have an abscess or cellulitis than those who did not. A study of 40 hospitalised PWID in New York found that 33% (*n* = 13) reported licking their needles prior to injection with three also licking their skin prior to injection to clean the site and 12 afterwards [45]. The majority 68% (*n* = 27) had been admitted to hospital for treatment of bacterial infections although no statistically significant association was found between needle licking and bacterial infection. Several case series and reports have documented infections (primarily endocarditis, but also femoral abscess) attributed to oral bacterial (oropharyngeal organisms) among PWID who report licking their needle and/or injection site prior to drug injection [46–49].

Of note, is the consistency of detail provided by qualitative participants about preparation of heroin and crack cocaine in combination for injection and that this differs from the only other study found to illustrate the steps taken in this process. Ponton and Scott (2004) recreated typical injection preparation practices with 65 people recruited through three NSP sites in England. Injection preparation was demonstrated for the following: heroin (*n* = 47), crack cocaine (*n* = 8) and heroin and crack together (*n* = 10). In contrast to Ponton and Scott’s findings that crack is added to the heroin mixture after heating, our participants all describe using cold water (or water alternative) to cool down the prepared heroin solution before crack addition. This is undertaken to prevent the crack cocaine congealing in a high temperature solution but may also have implications for bacterial contamination and infection. Heating drug solutions has been indicated to inactivate HIV [50] and prevent systemic candidal infections [51]. Heroin solutions in the UK are prepared with acid to convert base heroin to a soluble salt. The excess use of acid is implicated in the causal chain to SSTI by precipitating venous sclerosis [35, 52]. Heating with acid can, however, also kill non-spore-forming bacteria, depending on the solution acidity and temperature reached.

It is difficult to assess bacterial risks associated with the use of cold water for injection preparation or the use of alternative water sources, given that most literature mentioning water in the injection process does so in the context of HIV and HCV transmission potential. Ciccarone and Bourgois [53] present a hypothesis that cold water heroin solutions may have fostered HIV transmission in the early phases of the epidemic in the USA. More recently, a model of HIV transmission examining heroin source-type (including cold water prepared solutions) supports this prior hypothesis [54]. Gaskin et al. [55] present one of the few studies with a HCV transmission remit to detail water source. Participants comprise 40 NSP clients based in Worcestershire, England. The majority (70%) reported using one water source only: for 72%, this was tap water, followed by 21% using boiled and cooled water. The authors highlight the discrepancy between participant-reported perceptions of safe (or ideal) practice and their actual practice. While the majority used tap water for injection preparation, only 10% considered this to be an ideally hygienic source. The authors make a strong recommendation for provision of sterile water for injection, in order to “meet the basic needs of individuals and help facilitate behavioural change” [55:429]. They report that, subsequent to their study, the Worcestershire Community Drug Team commenced the low-threshold provision of sterile water in ampoules.

Ponton and Scott (2004), also recruiting PWID through NSP in England, include information on water source. Of the 57 participants demonstrating heroin preparation, with or without crack cocaine, 61% (*n* = 35) used tap water, with other sources including bottled water (18%, *n* = 10), boiled water (11%, *n* = 6) and sterile water from ampoules (2%, *n* = 1). The only group documented not to heat their solution were people preparing crack on its own, with three using tap water, one using “boiled and cooled” water from the kettle, one using bottled water and one using water obtained from a toilet. Despite the latter finding, no reflection is included on the bacterial or other health implications of this practice. Interventions may be feasible in reducing risk. Campbell [23] reports on the pilot provision of 2 ml plastic water ampoules to PWID in Glasgow over 2 months in 2012. Prior to the pilot, only three of 74 NSP services provided WFI, and this was in glass ampoule form. The plastic ampoules were supplied separately to the injecting packs, with PWID invited to take as many as they needed. Over the 2 months, at the three pilot sites, 31,518 ampoules were distributed, with footfall at the sites increasing by 25% during this period compared to the preceding 2 months. Sites not providing water experienced a 5% decrease. Of 42 clients interviewed during this time, four (9%) identified using toilet water (from the bowl) or puddle water prior to the pilot—with none reporting use of these sources during the intervention.

Concerns around the potential for HCV transmission through sharing of water among PWID led to the legal framework for UK supply described in the introduction. Given the reluctance to provide glass ampules by some local authorities, it is timely to revisit this evidence and associated guidance. Heimer and colleagues [56] report recent laboratory studies illustrating that HCV transmission occurs via the needles and syringes used in preparing and injecting drugs, not through shared paraphernalia. They recommend, given these results, that NSP “may want to reconsider expanding scarce resources to provide supplies that will do little or nothing to prevent HCV transmission” [56:471]. This view surprises in its blindness to risks other than viral transmission that could be obviated through the provision of sterile injecting paraphernalia. The reuse of stored filters for injecting, for example, is likely to pose a bacterial and fungal infection risk. Although bacterial risks from using non-sterile and alternative water sources are not well documented, our qualitative data in combination with the high prevalence of SSTI and related hospitalisation among the survey sample indicate a need for policy reform. We call for funding and increased supply of adequate quantities of WFI to people who do not have access to clean water. Greater awareness of the legality of 5 ml WFI provision for PWID is likely to facilitate distribution, given that these are marketed at a similar cost to 2 ml glass ampoules. Our study finding dissemination groups with PWID indicate that 5 ml WFI are desired, but that some sharing would take place. Despite evidence of reduced HCV transmission risk through water, provision of small volume (2 ml) plastic ampoules (licensed but not currently marketed, and therefore costly) would be ideal. Relaxation of prescription only medicine regulations that prevent drug services and other non-pharmacy suppliers splitting WFI packs (10 ampules) into smaller units would remove an additional important barrier to adequate distribution.

**Fig. 1:**
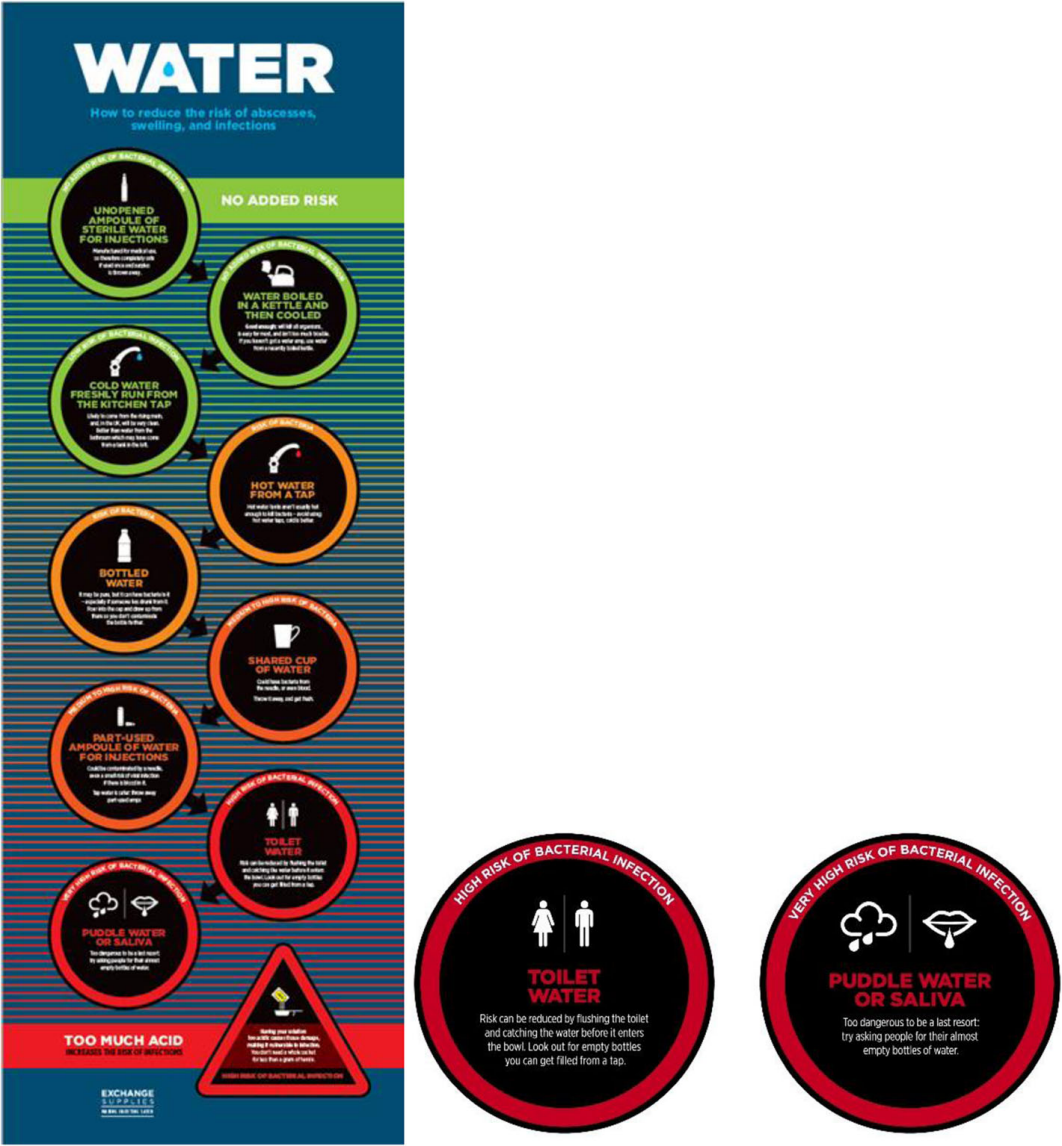
Water risks poster – Exchange Supplies (2019)

The use of unsafe alternatives to WFI potentiates high health risks and health system burden among an increasingly vulnerable population of unstably housed PWID. We call, therefore, not only for policy reform but also for a revolution in harm reduction messaging, whereby “best practice” is presented as just one option among many—with others offered tailored to different levels of environmental constraint. In a context whereby PWID report use of puddle water, alcohol and saliva to prepare drugs for injection, harm reduction messaging that enjoins people to “wash their hands” before injecting, alongside the provision of injecting kits without water, enacts a structural violence—shaming through a denial of the constraints faced by the most marginalised. Here, disconnect between drug treatment providers, policy makers and those who they are served to care for is profound. Messaging based on a hierarchy of safety provides an alternative to the alienating potential of “hyper sanitary” [57] best practice. In response to the findings of the Care & Prevent study, Exchange Supplies (a social enterprise in the UK that provides harm reduction equipment and materials) has adapted its water risks poster for display in NSP (Fig. 1). In addition, the wider environmental constraints placed on actioning such advice need to be considered. More widespread distribution of WFI from NSP is required, alongside the wider provision of methods to clean hands and injecting sites. This is particularly crucial given the recent COVID-19 pandemic and constraints faced by the most marginalised to enacting protective practices. Installation of public water fountains, alongside the provision of handheld water purifiers and similar devices to homeless people who inject drugs, could also be considered as an environmentally sound way to provide water for skin washing and injection preparation practices.

**Fig. 2:**
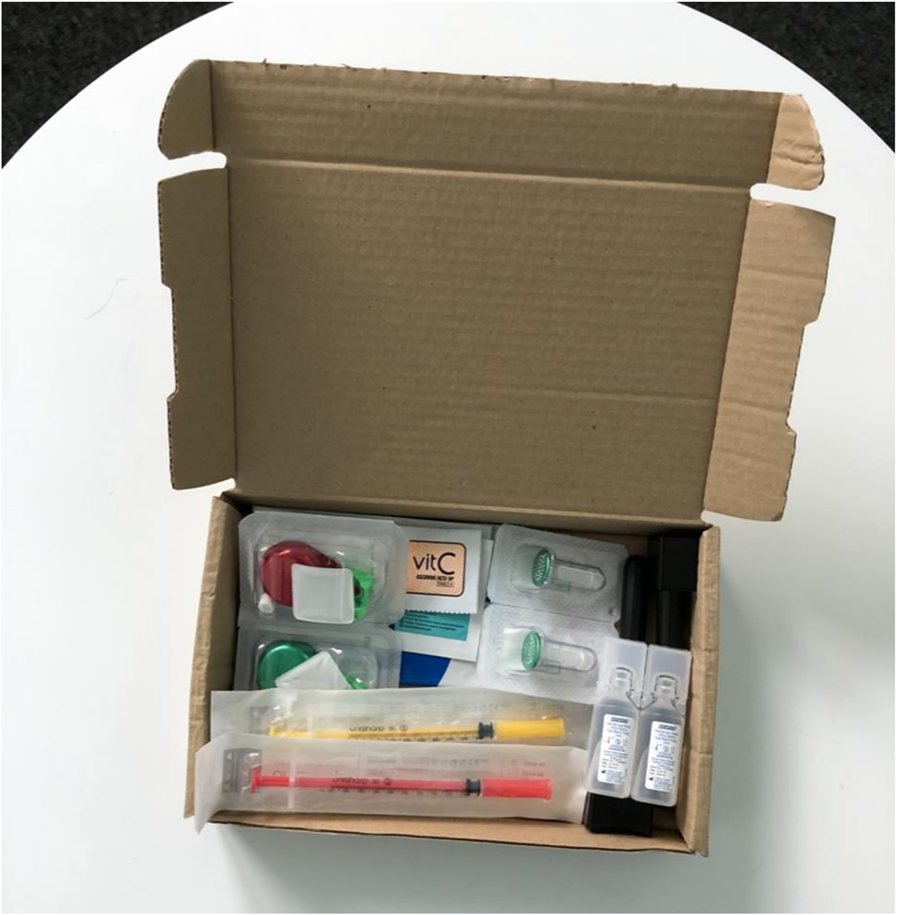
Street injecting kit – Exchange Supplies (2020)

In the time this paper has been in review, we have developed, in collaboration with Exchange Supplies, a prototype street injecting kit (Fig. 2). This responds to cost constraints to widespread WFI provision and the need identified through our work specifically for people who prepare and inject drugs in public spaces for access to water. The kit comes in a box that doubles as a stable safe injecting space with windbreak. Contents comprise two 30G syringes, four swabs, two Sterifilts, two vitamin C sachets, two spoons, two “fitstick” syringe disposal devices, two antimicrobial handwipes in a sachet and two 5 ml sterile water ampoules. The addition of antimicrobial handwipes attends to difficulties people have in accessing running water for washing hands and is of added value in relation to COVID-19 and other infection prevention for PWID—many of whom have compromised immune and respiratory systems.

## Conclusion

Our study highlights the role of unsafe liquids used to prepare drug injection solutions. Given the serious health harms associated with this practice, it is crucial that services engage with the everyday realities of people living on the streets and amend service provision accordingly. Current harm reduction messaging and injecting equipment provision in the UK is failing to meet the needs of the most marginalised. There is an urgent need to revisit the marketing and/or legal limits to the volume of WFI ampoules supplied to PWID, particularly given that concerns about sharing water increasing HCV transmission seem not to have been borne out in subsequent analysis.

## Data Availability

The datasets used and/or analysed during the current study are available from the corresponding author on reasonable request.

## Abbreviations

COPD: Chronic obstructive pulmonary disease
COVID-19: Coronavirus
EU: European Union
HCV: Hepatitis C
MHRA: Medicines and Healthcare Regulatory Authority
NSP: Needle and syringe programmes
ODK: Open Data Kit
PWID: People who inject drugs;
SSTI: Skin and soft tissue infections
UK: United Kingdom
WFI: Water for injection

## Footnotes

1 The participant’s reference to ‘mls’ is to the unit grading on an insulin syringe, with 100 units equivalent to 1 ml.

2 London is base for 27% of people who are street homeless in England. This is a growing population, with street counts and estimates placing 1,283 people rough sleeping in London on one Autumn night in 2019, representing a 13% increase from a comparable night in 2017 [41].
