# Correction to: Navigating environmental constraints to injection preparation: the use of saliva and other alternatives to sterile water among unstably housed PWID in London

**DOI:** 10.1186/s12954-020-00388-x

**Published:** 2020-06-09

**Authors:** Magdalena Harris, Jenny Scott, Vivian Hope, Talen Wright, Catherine McGowan, Daniel Ciccarone

**Affiliations:** 1 Department of Public Health, Environments and Society, London School of Hygiene & Tropical Medicine, 15-17 Tavistock Place, London, WC1H 9SH UK; 2 Department of Pharmacy & Pharmacology, University of Bath, Claverton Down, Bath, BA2 7AY UK; 3 Public Health Institute, Liverpool John Moores University, Tithebarn Street, Liverpool, L2 2QP UK; 4 School of Medicine, University of California, San Francisco, 513 Parnassus Ave, San Francisco, CA 94143-0410 USA

**Correction to: Harm Reduction Journal (2020) 17:24**

**https://doi.org/10.1186/s12954-020-00369-0**

Following publication of the original article [1], the authors would like to correct two sentences in the article:

'Drug-related deaths in the UK are higher than any European Union (EU) country; in the year before Britians exit they accounted for one third of all reported in the EU country and, in the year prior to Britain’s exit, accounted for a third of all reported in the EU'

should read: 'Drug-related deaths in the UK are higher than any European Union (EU) country; in the year prior to Britain’s exit, they accounted for a third of all reported in the EU'.

and

‘Current harm reduction advice advocates the use of WFI, with the “next best” alternative boiled and cooled potable (drinkable cold tap) water, and after this, cold tap water’.

should read: ’Current harm reduction advice advocates the use of WFI, with the “next best” alternative boiled and cooled potable (drinkable) tap water, and after this, cold tap water’.
